# Metabolic Characteristics of a Glucose-Utilizing *Shewanella oneidensis* Strain Grown under Electrode-Respiring Conditions

**DOI:** 10.1371/journal.pone.0138813

**Published:** 2015-09-22

**Authors:** Gen Nakagawa, Atsushi Kouzuma, Atsumi Hirose, Takuya Kasai, Gen Yoshida, Kazuya Watanabe

**Affiliations:** School of Life Sciences, Tokyo University of Pharmacy and Life Sciences, Tokyo, Japan; University of Houston, UNITED STATES

## Abstract

In bioelectrochemical systems, the electrode potential is an important parameter affecting the electron flow between electrodes and microbes and microbial metabolic activities. Here, we investigated the metabolic characteristics of a glucose-utilizing strain of engineered *Shewanella oneidensis* under electrode-respiring conditions in electrochemical reactors for gaining insight into how metabolic pathways in electrochemically active bacteria are affected by the electrode potential. When an electrochemical reactor was operated with its working electrode poised at +0.4 V (*vs*. an Ag/AgCl reference electrode), the engineered *S*. *oneidensis* strain, carrying a plasmid encoding a sugar permease and glucose kinase of *Escherichia coli*, generated current by oxidizing glucose to acetate and produced D-lactate as an intermediate metabolite. However, D-lactate accumulation was not observed when the engineered strain was grown with a working electrode poised at 0 V. We also found that transcription of genes involved in pyruvate and D-lactate metabolisms was upregulated at a high electrode potential compared with their transcription at a low electrode potential. These results suggest that the carbon catabolic pathway of *S*. *oneidensis* can be modified by controlling the potential of a working electrode in an electrochemical bioreactor.

## Introduction

The environmental redox state is an important factor determining the growth and metabolic activities of microorganisms because it affects the availability of electron acceptors and cellular energy conservation processes, such as respiration and fermentation [1]. In the respiratory chain, the difference in redox potentials between electron donors and acceptors determines the amount of energy conserved during electron transfer reactions. The intracellular redox balance, which influences fermentative products, is also associated with the environmental redox state [2]. In biotechnological processes, the amount of electron acceptors (e.g., oxygen) is used for controlling the production rate and target compound yields [3].

Recent studies have suggested that bioelectrochemical systems (BES) are useful for controlling microbial metabolic activities [4]. BES are biotechnological systems that utilize the association between electrodes and electrochemically active bacteria (EAB). BES have attracted considerable attention because of their wide applicability for valuable biotechnological processes, including electricity generation (i.e., microbial fuel cells; MFC) [5] and the production of fuels and chemicals (i.e., microbial electrosynthesis) [6]. In BES, the redox state of electron acceptors (or donors) and rate of electron flow during metabolism can be altered by controlling the electrode potential, allowing modification of the intracellular redox balance in electrode-associated microbes [6,7]. Studies have also demonstrated that differences in electrode potential and/or catalytic current in BES influence the gene expression profiles of EAB [8–10]. To cite an example, Matsuda et al. [10] have reported that the expression levels of the genes associated with the TCA cycle in *Shewanella loihica* PV-4 were markedly altered when cells were cultivated in the presence of electrodes poised at different potentials. However, limited information is available on how EAB control metabolism and alter their intracellular metabolic products in response to changes in the electrode potential.

*Shewanella oneidensis* MR-1 is one of the most extensively studied EAB, owing to its annotated genome sequence [11], ease of genetic manipulation [4], and capability to transfer electrons to extracellular electrodes without an exogenously added mediator [12]. Further, recent studies have demonstrated the potential applicability of this strain for microbial electrosynthesis systems [13]. Because MR-1 has also been well characterized in terms of its central carbon utilization pathways [14–19], it is considered suitable as a model bacterium for studying the metabolic characteristics of EAB during electrochemical cultivation in BES. However, studies have also revealed that MR-1 and many other *Shewanella* strains preferably utilize low-molecular-weight organic acids, including lactate and pyruvate, as carbon and energy sources. They do not prefer five- and six-carbon carbohydrates, including glucose, although these sugars are widely used as substrates for MFC and bioproduction processes. Therefore, metabolic engineering of MR-1 to confer the ability to utilize glucose may be valuable not only for expanding the applicability of this strain for biotechnological applications but also for understanding how EAB metabolize carbohydrates under potential-controlled conditions.

Spontaneous and engineered glucose-utilizing *S oneidensis* strains have been obtained in previous studies. Howard et al. [20] have reported that MR-1 acquired the ability to utilize glucose after an initial exposure to glucose. More recently, Choi et al. [21] successfully constructed glucose-utilizing *Shewanella* mutants by introducing the glucose facilitator (*glf*) and glucokinase (*glk*) genes from *Zymomonas mobilis*. However, although they also demonstrated that engineered MR-1 was able to generate current using glucose as the electron donor in an MFC reactor [21], the metabolic profiles of sugar-utilizing *Shewanella* strains during electrode respiration have not been investigated. In the present study, we constructed an engineered *S*. *oneidensis* strain by introducing glycolytic genes from *Escherichia coli*. We then characterized the glucose utilization profiles of this strain under electrode-respiring conditions in BES, with a particular focus on differences in D/L-lactate production from glucose and the expression levels of genes involved in pyruvate and lactate metabolism.

## Materials and Methods

### Bacterial strains and growth conditions

*S*. *oneidensis* MR-1 was obtained from the American Type Culture Collection (ATCC). *E*. *coli* strains [22] were routinely cultured in Luria–Bertani (LB) medium at 37°C. The *E*. *coli* mating strain (WM6026) required supplementation of the medium with 100 μg/ml 2,6-diaminopimelic acid (DAP) for growth. *Shewanella oneidensis* strains were cultured at 30°C in LB or in lactate minimal medium (LMM) [23] containing 15 mM lactate as the carbon source and supplemented with 0.2 g/l casamino acids, 10 ml/l of each amino acid, and 10 ml/l of each trace mineral solution. Glucose minimal medium (GMM), which contained 10 mM or 15 mM glucose instead of the lactate in LMM, was used for cultivation with glucose as the carbon and energy source. For aerobic cultivation, *S*. *oneidensis* strains were introduced into 300-ml baffled Erlenmeyer flasks containing 100 ml LMM or GMM, and were cultivated with shaking on a rotary shaker at 180 rpm. For anaerobic cultivation, *S*. *oneidensis* strains were introduced into 100-ml bottles containing 80 ml GMM supplemented with 5 mM or 40 mM fumarate. The bottles containing the anaerobic cultures were capped with Teflon-coated butyl rubber septa, sealed with aluminum crimp seals, and purged with pure nitrogen gas. The optical densities at 600 nm (OD_600_) of the cultures were measured using a DU800 spectrophotometer (Beckman). When necessary, 15 μg/ml gentamicin (Gm) was added to the culture media. Agar plates contained 1.6% Bacto agar (Difco).

### Mutant construction

The *glk* (ECK2384) and *galP* (ECK2938) genes were amplified from the genomic DNA of an *E*. *coli* K-12 derivative, strain DH5α, using primer sets glk-F-KpnI and glk-R-XhoI, and galP-F-XhoI and galP-R-PstI, respectively (see S1 Table in the Supporting Information). The PCR products obtained were digested by the restriction enzymes corresponding to the sites incorporated in the primers (XhoI and either KpnI or PstI) and then ligated into KpnI-PstI-digested pBBR1MCS-5 [24]. The resultant plasmid, pBBR-*glk*-*galP*, was introduced into *S*. *oneidensis* cells by filter mating with *E*. *coli* WM6026.

In-frame disruption of the *dld-II* and *ldhA* gene in strain MR-1 was performed using a two-step homologous recombination method with suicide plasmid pSMV-10, as described previously [22,23,25]. Briefly, a 1.6-kb fusion product, consisting of upstream and downstream sequences of the *dld-II* or *ldhA* gene joined by an 18-bp linker sequence, was constructed by PCR and *in-vitro* extension using the primers listed in S1 Table. The amplified fusion product was ligated into the SpeI site of pSMV-10, generating pSMV-dld-II or pSMV-ldhA, which was then introduced into MR-1 by filter mating with *E*. *coli* WM6026. Transconjugants (single-crossover clones) were selected on LB plates containing 50 μg/ml kanamycin (Km) and were further cultivated for 20 h in LB medium lacking antibiotics. The cultures were then spread onto LB plates containing 10% (w/v) sucrose to isolate Km-sensitive double-crossover mutants. Disruption of the target gene in the obtained strains was confirmed by PCR. One representative mutant strain in which the *dld-II* or *ldhA* gene was disrupted in-frame was selected and designated *Δdld-II* or Δ*ldhA*, respectively. To construct a *dld-II*/*ldhA* double-knockout mutant (Δ*dld-II*Δ*ldhA*), pSMV-ldhA was introduced into the Δ*dld-II* cells, and the double-crossover mutants were screened as described above.

### Operation of electrochemical cells

A small double-chambered EC (36 ml total capacity) was used to monitor and compare currents generated by *S*. *oneidensis* strains. This EC was equipped with a graphite felt working electrode (3.0 cm^2^; poised at +0.4 V *vs*. Ag/AgCl) and an Ag/AgCl reference electrode (HX-R5, Hokuto Denko) in the anode chamber and a platinum wire counter electrode (5 cm, φ0.3 mm; Nilaco) in the cathode chamber. A Nafion 117 proton-exchange membrane (7.1 cm^2^; Sigma-Aldrich) was used to separate the anode and cathode chambers. The anode chamber was filled with 15 ml of LMM or GMM that had been supplemented with 4 mM lactate or 2 mM glucose as the electron donor and 170 mM NaCl as an electrolyte, and then inoculated with bacterial cells at an initial OD_600_ of 0.01. The cathode chamber was filled with 15 ml of the same medium without glucose. Current was monitored using a multichannel potentiostat (VPM3; Biologic), and a current density (μA/cm^2^) was calculated based on the projected area of working electrode (3.0 cm^2^). Coulombic efficiency was calculated by dividing the total number of electrons transferred to the working electrode by the theoretical maximum number of electrons produced by complete substrate oxidation to CO_2_ (24 e^–^/mol for glucose and 12 e^–^/mol for lactate).

A large double-chambered EC (360 ml total capacity) was used for metabolite and transcriptional analyses of MR-1(pBBR-*glk*-*galP*). This EC was equipped with a graphite felt working electrode (8 cm^2^) and an Ag/AgCl reference electrode in the anode chamber and a platinum wire counter electrode (10 cm) in the cathode chamber. A Nafion 117 proton exchange membrane (28 cm^2^) was used to separate the anode and cathode chambers. The anode chamber was filled with 150 ml of GMM supplemented with 2 mM glucose and 170 mM NaCl, and then inoculated with bacterial cells at an initial OD_600_ of 0.01. The cathode chamber was filled with 150 ml of the same medium without glucose. A current density (μA/cm^2^) was calculated based on the projected area of working electrode (4.0 cm^2^). Reproducibility was assessed using at least three independent measurements, and typical data are shown here. For metabolite analyses, the working anode electrode was poised at 0 V or +0.4 V (*vs*. Ag/AgCl) using a VPM3 potentiostat. For transcriptional analyses, the working electrode was poised at 0 V, and then after the electric current became stable, the electrode potential was changed to +0.3 V or –0.3 V. After further cultivation in the ECs for 2 h, the bacterial cells attached to the working electrode were collected and subjected to RNA extraction.

### Metabolite analyses

After the cells were removed by filtration through a membrane filter unit (0.20 μm pore size, DISMIC-25HP; Advantec), the amounts of acetate, formate, and some other organic acids in the EC supernatant were measured using a previously described high-performance liquid chromatography (HPLC; Agilent 1100 series) method [22]. Glucose and D/L-lactate in the filtered supernatant were measured with enzymatic assays that were performed using an F-kit (J. K. international) according to the manufacturer’s instructions.

### RNA extraction

Total RNA was extracted using Trizol reagent (Invitrogen) according to the manufacturer’s instructions and subsequently purified using an RNeasy Mini Kit and an RNase-Free DNase Set (Qiagen). The quality of extracted RNA was evaluated using an Agilent 2100 Bioanalyzer with RNA 6000 Pico reagents and RNA Pico Chips (Agilent Technologies) according to the manufacturer’s instructions.

### Quantitative RT-PCR

Quantitative RT-PCR (qRT-PCR) was performed using a LightCycler 1.5 instrument (Roche) according to a previously described method [26–28]. The PCR mixture (20 μl) contained 15 ng total RNA, 1.3 μl of 50 mM Mn(OAc)_2_ solution, 7.5 μl LightCycler RNA Master SYBR Green I (Roche), and 0.15 μM of the primers listed in S1 Table in the Supporting Information. To generate standard curves, DNA fragments from the target genes *dld*-*II*, *lldF*, *ldhA*, *aceF*, *pykA*, *eda*, *pta*, *pflB*, and the 16S rRNA gene were amplified by PCR using the total DNA of strain MR-1 as the template. These DNA fragments were subsequently purified by agarose gel electrophoresis using a QIAEX II Gel Extraction Kit (Qiagen). A dilution series of the purified products from each PCR reaction and the original RNA samples were used as templates for qRT-PCR analysis. Specificity of the qRT-PCR was verified by dissociation-curve analysis. The expression levels of the target genes (*dld*-*II*, *lldF*, *ldhA*, *aceF*, *pykA*, eda, *pta*, and *pflB*) were normalized based on the expression level of the reference gene (16S rRNA gene). All measurements were performed in triplicate at a minimum, and the data were statistically analyzed by the Student’s t-test. A *P* value of 0.01 was considered statistically significant.

## Results and Discussion

### Construction of glucose-utilizing mutant

**Fig 1:**
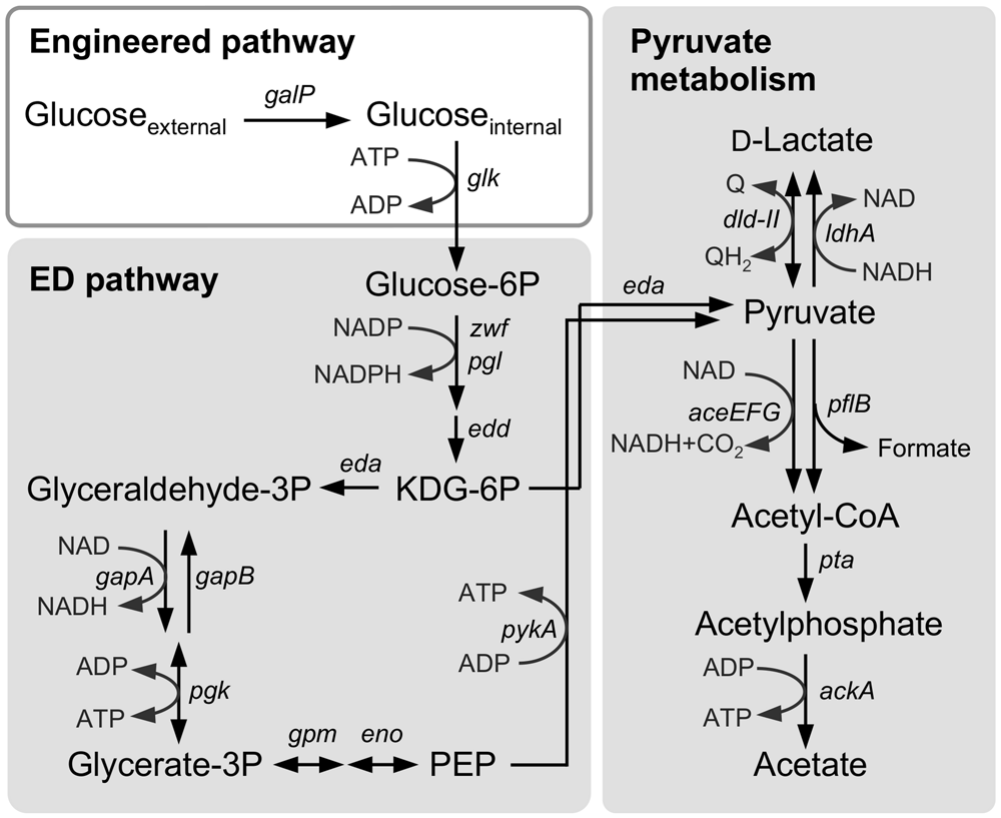
The glycolytic pathway in the engineered *S*. *oneidensis* MR-1. The engineered pathway constructed in this study is shown in a white box. Intrinsic catabolic pathways shown in shaded boxes are depicted based on findings reported in the literature [15,17–19,35].

The annotated genomic sequence data of *S*. *oneidensis* MR-1 (Genbank accession no. AE014299) suggest that this strain is able to metabolize glucose-6-phosphate and its downstream glycolytic metabolites through the Entner–Doudoroff (ED) and pentose phosphate (PP) pathways. However, this strain is unable to take up and phosphorylate glucose because a frameshift exists in the glucose/galactose transporter gene, *gluP* (SO_2214) [16,29], and no glucokinase gene (*glk*) can be found in the genome. Although MR-1 has a complete set of genes encoding the phosphoenolpyruvate (PEP):sugar phosphotransferase system for glucose (PTS^Glc^; *ptsHI-crr* and *ptsG*), it is known that this system does not support the growth of bacteria on glucose through the ED and PP pathways. This is because these glycolytic pathways cannot produce a sufficient amount of PEP for the phosphotransferase reaction of the PTS [16]. However, previous studies have reported that the introduction of *glk* and a glucose/galactose-proton symporter gene (*galP*) restored the ability of PTS^Glc^-inactivated *E*. *coli* mutants to utilize glucose by allowing glucose uptake and phosphorylation without the consumption of PEP [30–32]. In the present study, we constructed plasmid pBBR-*glk*-*galP*, which contains the *glk* and *galP* genes derived from *E*. *coli* K-12, and introduced it into MR-1 to confer the ability to utilize glucose on this strain (Fig 1).

**Fig 2:**
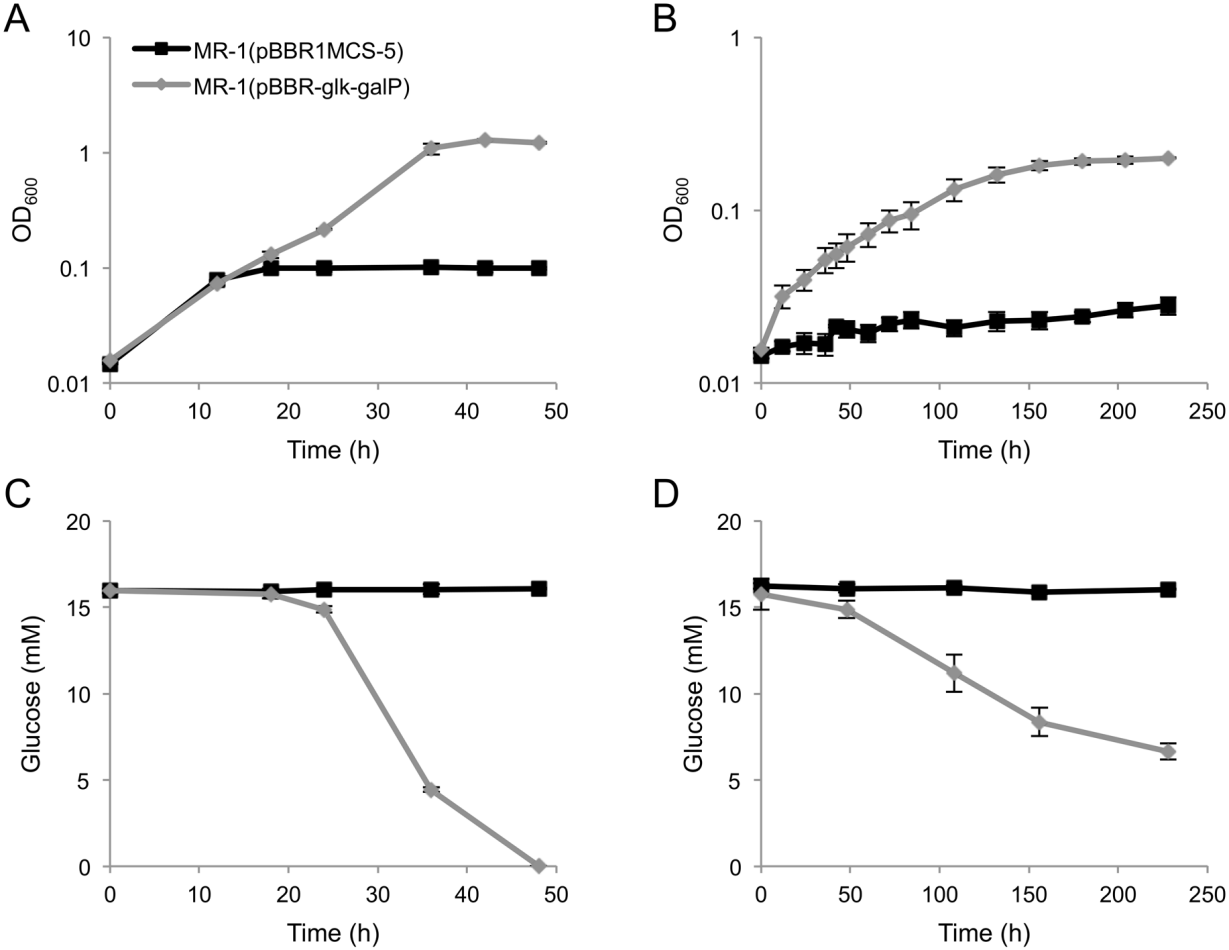
Growth (A, B) and glucose consumption (C, D) of *S*. *oneidensis* derivatives under aerobic (A, C) and fumarate-reducing (B, D) conditions. *S*. *oneidensis* cells harboring pBBR1MCS-5 (control vector) or pBBR-*glk*-*galP* were grown in GMM-containing 15 mM glucose as the electron acceptor. Anaerobic cultures were supplemented with 40 mM fumarate as the electron donor. Error bars represent standard deviations calculated from triplicate measurements.

When MR-1(pBBR-*glk*-*galP*) cells were cultivated in GMM under aerobic or anaerobic, fumarate-reducing conditions, cell growth accompanied by glucose consumption was observed (Fig 2A to 2D), demonstrating that introduction of these *E*. *coli* glycolytic genes allowed MR-1 to acquire the ability to grow on glucose. However, when the cells were incubated under anaerobic condition in the absence of any electron acceptor, substantial cell growth was not observed within the first 5 days of incubation (data not shown). This indicates that the engineered strain cannot acquire sufficient energy for growth under these fermentation conditions. A similar result has also been observed for another engineered strain of *S*. *oneidensis* [21], which exhibited only very low biomass production even when cells were cultivated for 18 days under glucose-fermenting conditions. The poor growth of the MR-1 derivatives during glucose fermentation is likely related to the low ATP yields that result from glycolysis through the ED pathway [33].

### Current generation by MR-1(pBBR-*glk*-*galP*)

**Fig 3:**
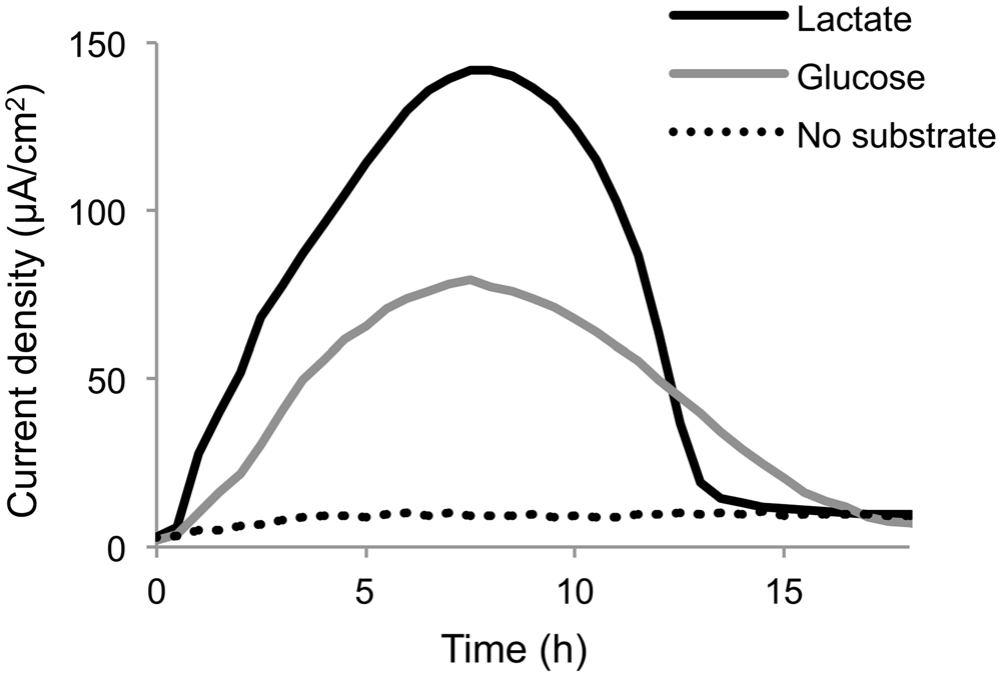
Current generation from glucose or lactate by MR-1 (pBBR-*glk*-*galP*). Cells were introduced into ECs supplemented with a minimal medium containing lactate or glucose as the electron donor and grown in the presence of a working electrode poised at +0.4 V (*vs*. Ag/AgCl). Results represent means of at least two parallel but independent experiments.

Current generation from glucose by MR-1(pBBR-*glk*-*galP*) was analyzed and compared with that from lactate using a small double-chambered EC equipped with a working electrode poised at +0.4 V (*vs*. Ag/AgCl) (Fig 3). The results demonstrate that the engineered strain is able to generate current using glucose as the electron donor. However, the maximum current density obtained from glucose (77.3 μA/cm^2^) was 55% lower than that obtained from lactate (140 μA/cm^2^), suggesting that the rate of glucose metabolism in the engineered MR-1 was lower than the rate of lactate metabolism. This difference in the current densities may reflect a difference in the growth rates of this strain in GMM and LMM (0.014 h^–1^ and 0.35 h^–1^, respectively, under fumarate-reducing conditions). Coulombic efficiencies in glucose- and lactate-supplemented ECs were calculated to be 10.3% and 19.3%, respectively, indicating that many of the supplemented substrates were not completely oxidized in these ECs. Previous studies have reported that *Shewanella* strains exhibits low coulombic efficiencies in lactate-supplemented MFC because they partially oxidize lactate and produce acetate as the major metabolite [22,34]. Similarly, in the present study, acetate was detected at concentrations of 2.3 mM and 2.9 mM in the supernatant of the glucose- and lactate-supplemented ECs, respectively, when the substrates were completely consumed (18 h after commencing the incubation). The molar yields of acetate from glucose and lactate were 58% and 73%, respectively. These results indicate that current generation from glucose by the engineered MR-1 mainly occurs through partial substrate oxidation of glucose to acetate, as is the case for current generation from lactate.

### Metabolic responses to different electrode potentials

**Fig 4:**
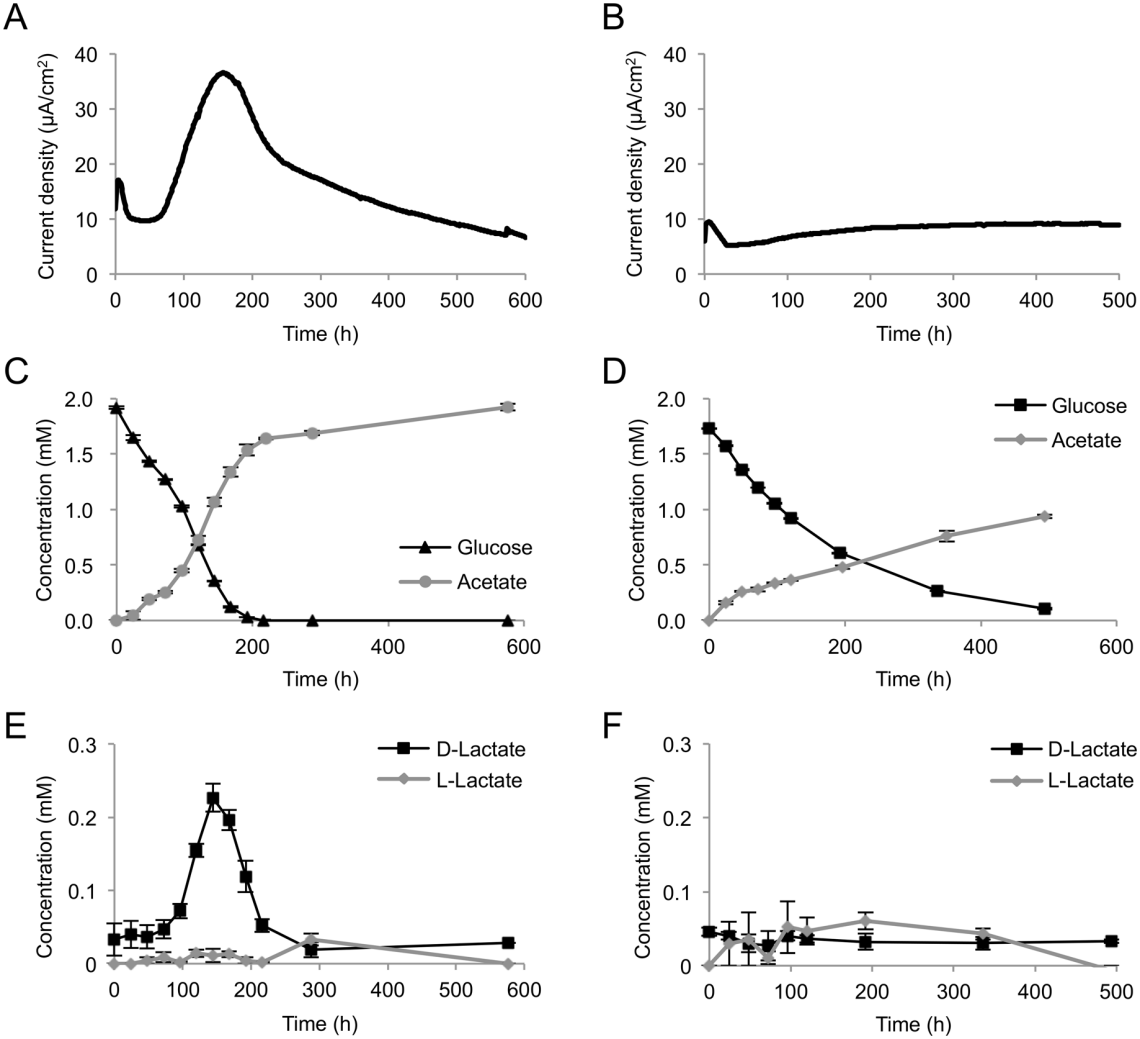
Current (A, B) and metabolite (C, D, E, F) production from glucose by MR-1(pBBR-*glk*-*galP*) in ECs operated with HP (A, C, E) or LP (B, D, F) electrode. Cells were cultivated in ECs supplemented with 2 mM glucose as the electron donor. The error bars represent the standard deviations calculated from triplicate measurements.

To investigate the influence of electrode potentials on the metabolic activity of engineered *S*. *oneidensis*, current generation and metabolite production from glucose were monitored using an EC equipped with a working electrode poised at +0.4 V (*vs*. Ag/AgCl) (high potential, HP) or 0 V (low potential, LP) (Fig 4A to 4F). In this experiment, we used a large double-chambered EC for stable sampling of supernatants from the anode chamber. Higher electric current and glucose-consumption rate were observed under the HP condition (Fig 4A and 4C) than were observed under the LP condition (Fig 4B and 4D). Although Matsuda et al. [10] have reported that *S*. *loihica* PV-4 generated decreased current when cells were grown in an EC equipped with a tin-doped, In_2_O_3_ (ITO)-coated glass working electrode poised at a high potential, such decreases in current at higher potentials were not observed in the present study. It is likely that differences in electrode materials and configuration of the EC affect the current-generation profiles of *Shewanella* strains under poised potential conditions.

Acetate was detected in ECs operated under the HP and LP conditions, along with current generation and glucose consumption (Fig 4C and 4D). Interestingly, a significant amount of D-lactate was detected, along with an increase in current density, under the HP condition (Fig 4E). This demonstrates that the engineered MR-1 was able to ferment glucose and produce D-lactate as an intermediate metabolite under electrode respiration. D-lactate was completely consumed within 300 h (Fig 4E), indicating that this metabolite was oxidized to acetate for current generation. However, under the LP condition, substantial quantities of D-lactate were not detected (Fig 4F). These results suggest that the difference in the electrode potential influences the glycolytic flux to D-lactate in the engineered *S*. *oneidensis*. L-lactate did not remarkably accumulate under HP or LP conditions (Fig 4E and 4F). Formate and other organic acids, including succinate, fumarate, propionate, and maleate, were not detected in this experiment (data not shown), while Choi et al. [21] have reported that a glucose-utilizing *S*. *oneidensis* strain produced formate in addition to acetate and lactate (the chirality was not identified in that experiment) during glucose metabolism in the presence or absence of Fe(III) oxide. This difference is likely due to the rapid oxidation of formate under poised electrode conditions.

### Identification of D-lactate-production pathways

**Fig 5:**
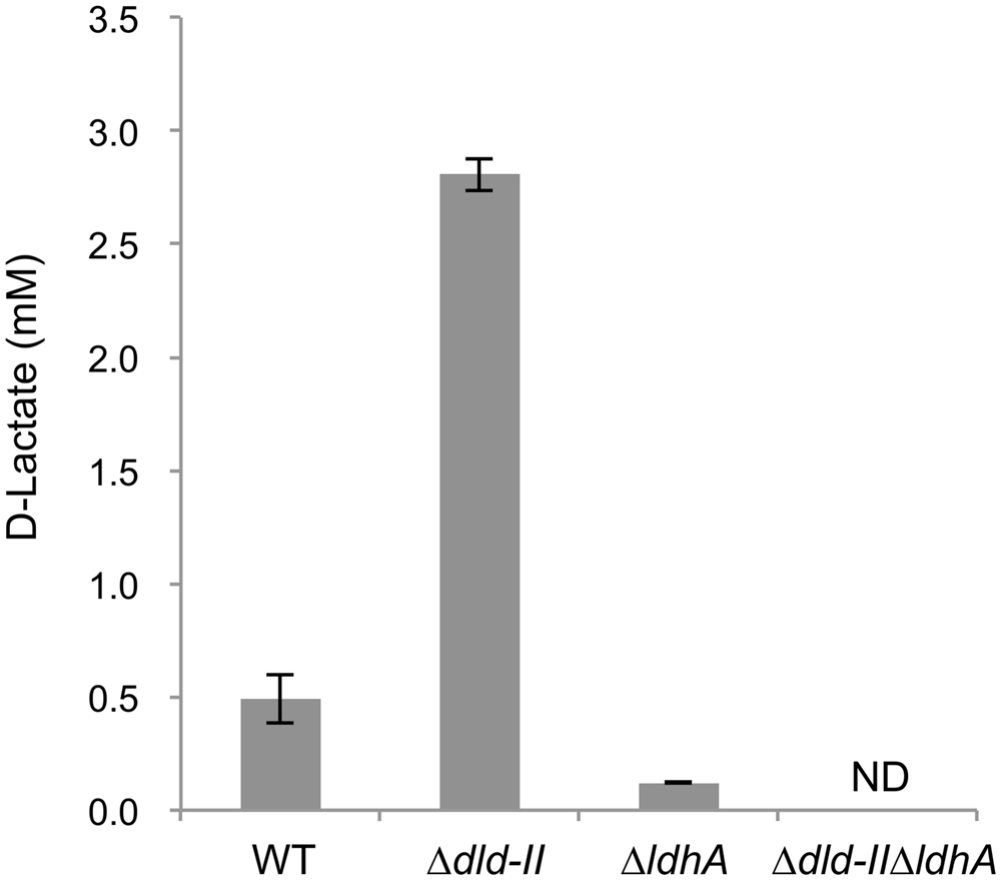
D-lactate production from glucose by D-LDH-deficient *S*. *oneidensis* derivatives. Cells were anaerobically grown in GMM containing 10 mM glucose and 5 mM fumarate until the electron acceptor was completely consumed (for 24 h). Error bars represent standard deviations calculated from triplicate measurements. ND, not detected (below detection limits; 0.02 mM).

Based on the annotated genomic sequence data, *S*. *oneidensis* MR-1 is predicted to have two D-lactate dehydrogenase (D-LDH) genes, i.e., *dld-II* (SO_1521) and *ldhA* (SO_0968). Previous studies have reported that, while Dld-II functions as the respiratory D-LDH required for D-lactate oxidation to pyruvate in MR-1 [15], this enzyme is also involved in pyruvate reduction to D-lactate during pyruvate fermentation [35]. LdhA belongs to a family of fermentative NADH-dependent D-LDHs [36], although the function has not yet been characterized in MR-1. To identify the gene(s) involved in D-lactate production in the engineered MR-1, we constructed two single-knockout mutants for these D-LDH genes (Δ*dld-II* and Δ*ldhA*) and a double-knockout mutant (Δ*dld-II*Δ*ldhA*), and examined their ability to produce D-lactate from glucose. In this experiment, wild-type MR-1 (WT), Δ*dld-II*, Δ*ldhA*, and Δ*dld-II*Δ*ldhA* cells transformed with pBBR-*glk*-*galP* were grown in GMM under electron acceptor (fumarate)-limited conditions, and D-lactate accumulation in culture supernatants was determined and compared (Fig 5). The results revealed that the production of D-lactate by Δ*ldhA* cells (0.122 ± 0.002 mM) was substantially lower than that by WT cells (0.493 ± 0.107 mM). Although Pinchuk et al. [35] reported that the deletion of *ldhA* in MR-1 did not impair lactate production during pyruvate fermentation, the above results indicate that LdhA functions as a major fermentative D-LDH during sugar utilization. However, Δ*ldhA* cells retained the ability to produce a small amount of D-lactate, while Δ*dld-II*Δ*ldhA* cells did not produce this metabolite at a detectable level (Fig 5). This observation indicates that Dld-II is partially involved in D-lactate production from glucose. We also found that Δ*dld-II* accumulated a higher concentration of D-lactate compared to WT (Fig 5), supporting that Dld-II is mainly involved in the oxidation of D-lactate to pyruvate. Taken together, these results indicate that, in the engineered glycolytic pathway, a substantial portion of pyruvate produced during glucose fermentation is converted to D-lactate by LdhA, and partly by Dld-II, although the product is largely reconverted to pyruvate by Dld-II (Fig 1).

### Expression of lactate and pyruvate metabolism genes

**Fig 6:**
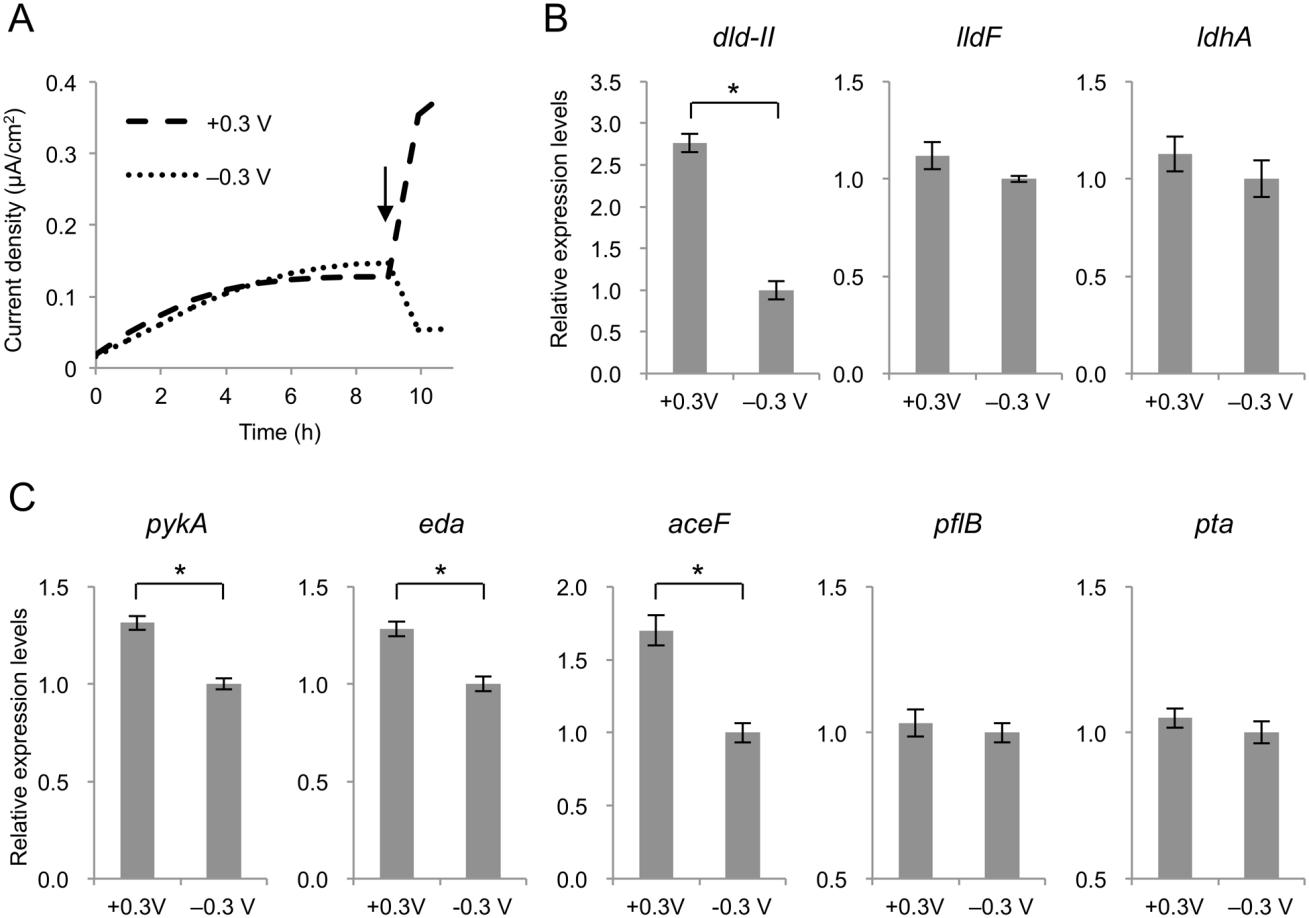
Current generation (A) and expression levels of the genes involved in lactate (B) and other carbon (C) metabolism under potential-controlled conditions. MR-1(pBBR-*glk*-*galP*) cells were cultivated in ECs containing media supplemented with 2 mM glucose as the electron donor. The arrow indicates the time point at which the electrode potential was shifted. Gene expression levels were analyzed by quantitative RT-PCR analysis. Error bars represent standard deviations calculated from at least three measurements. Astarisks indicate statistically significant differences (*P* < 0.01).

To explore the reason for the electrode potential-dependent accumulation of D-lactate, we investigated the transcriptional levels of the genes involved in lactate and pyruvate metabolism in cells grown at different electrode potentials. In this experiment, MR-1(pBBR-*glk*-*galP*) cells were cultivated at a poised electrode potential of 0 V until the electric current became stable (for 9 h), and then the potential was altered to +0.3 V or –0.3 V. This experiment confirmed that electric current was steeply increased or decreased by the shift in the electrode potential (Fig 6A). To investigate the influence of shifts in electrical potential on gene expression without substantial changes in metabolite concentrations, cells attached to the working electrodes were collected 2 h after the potential shifts. Total RNA extracted from these cells was analyzed using qRT-PCR. Expression levels of D- and L-LDH genes (*dld-II*, *ldhA*, and *lldF*) are shown in Fig 6B. Interestingly, the expression levels of *dld-II* were significantly increased with the increase in the electrode potential, while those of *ldhA* and *lldF*, which encodes a component of the respiratory L-LDH complex (LldEFG) [15], were not significantly affected by the potential shift (*P* < 0.01; Fig 6B). Potential-dependent expression was also observed for three genes involved in pyruvate metabolism, i.e., *pykA*, *eda*, and *aceF* (Fig 6C). The *pykA* (SO_2491) and *eda* (SO_2486) genes are annotated respectively as pyruvate kinase that catalyzes the conversion of PEP to pyruvate and 2-keto-3-deoxygluconate 6-phosphate (KDG-6P) aldolase that catalyzes the conversion of KDG-6P to pyruvate and glyceraldehyde 3-phosphate (see Fig 1). The *aceF* (SO_0425) gene is reported to encode a component of the pyruvate dehydrogenase (PDH) complex (AceEFG) involved in the conversion of pyruvate to acetyl-CoA and CO_2_ [35]. However, the expression of the pyruvate formate-lyase (*pflB*; SO_2912) and phosphotransacetylase (*pta*; SO_2916) genes was not significantly affected by the potential shift (*P* < 0.01; Fig 6C). These results suggest that the activity of key enzymes involved in pyruvate and D-lactate metabolism (i.e., PykA, PDH, and Dld-II; see Fig 1) is increased under HP conditions, although the mechanisms underlying these transcriptional changes and the accumulation of D-lactate are currently unclear. Since pyruvate oxidation to acetly-CoA by PDH involves the production of NADH, it is conceivable that the activation of PDH under HP conditions results in an increase in intracellular NADH, thereby enhancing the production of D-lactate catalyzed by LdhA. It is also possible that the activation of Dld-II contributes to a transient accumulation of D-lactate, as this enzyme catalyzes the bidirectional conversion between D-lactate and pyruvate (Fig 5). Further studies are underway to elucidate the complex carbon and electron fluxes for the D-lactate production under potential-controlled conditions.

Although reasons for the potential-dependent expression of the *dld-II*, *pykA*, *eda*, and PDH genes are currently unknown, it is conceivable that changes in intracellular redox states may influence the expression of these genes via redox-sensing regulators, such as PAS-domain containing proteins [1,37], as HP electrodes can act as efficient electron acceptors and promote the oxidation of intracellular molecules. Because the cultivation of MR-1 in the presence of D-lactate did not result in a significant increase in the expression of the *dld-II* and *ldhA* genes (S1 Fig), it is not likely that the accumulation of D-lactate under HP conditions induces the expression of these D-LDH genes. In contrast, previous studies have demonstrated that the expression of the L-LDH genes (*lldEFG*) requires LlpR (L-lactate-positive-regulator; SO_3460) [15,38], although the molecular mechanism underlying the positive regulation of *lldEFG* by this regulator remains unclear. As it has been reported that the transcription of L-LDH genes (*lldRDP*) in *E*. *coli* is regulated by the LldR regulator in a L-lactate-dependent manner [39], it is possible that the expression of the *lldEFG* genes in MR-1 is also regulated by the presence or absence of L-lactate. However, our data suggest that it is not linked to changes in extracellular or intracellular redox status.

## Conclusions

The results of the present study indicate that shifts in the electrode potential and concomitant changes in electric current in BES affect the glycolytic flux towards D-lactate production in the engineered glucose-utilizing MR-1. These results suggest that metabolite production via the central catabolic pathways in EAB can be modified through electrochemical approaches. The present results also reveal that the expression levels of the genes involved in pyruvate and D-lactate metabolism, including the PDH and *dld-II* genes, are dependent upon the electrode potential. Thus, it is possible to regulate the expression of key genes for target metabolite production through a combination of a potential-dependent transcriptional promoter and the electrode potential control. Although the accumulation of D-lactate observed in the present study was transient because of its oxidation to acetate during electrode respiration, genetic modification, such as the disruption of acetate synthesis genes and introduction of additional genes, would be applicable for the efficient production of valuable compounds. Ross et al. [13] have reported that *S*. *oneidensis* MR-1 can accept electrons from a low potential-poised electrode through the extracellular electron transfer pathway, suggesting the possibility that high value-added reductive products are synthesized by supplying electrons from the electrode to the engineered MR-1. We expect that the findings reported in the present study will be useful for the future development of BES-based biotechnology processes that will produce valuable chemicals.

## Supporting Information

S1 Fig qRT-PCR analyses of *dld-II* and *ldhA* in MR-1 cells grown with D-lactate and pyruvate. MR-1 cells were cultivated in LMM supplemented with 15 mM D-lactate as the carbon and energy source or in a pyruvate minimal medium containing 15 mM pyruvate (in substitution for lactate in LMM) up to the early stationary growth phase. Results are expressed as relative values to mRNA levels in cells grown on pyruvate. Error bars represent standard deviation calculated from at least three measurements.
(PDF)

S1 Table Primers used in this study. (PDF)

## References

[1] Green J, Paget MS. Bacterial redox sensors. Nat Rev Microbiol. 2004;2: 954–966. 15550941 10.1038/nrmicro1022

[2] Iuchi S, Weiner L. Cellular and molecular physiology of *Escherichia coli* in the adaptation to aerobic environments. J Biochem. 1996;120: 1055–1063. 9010748 10.1093/oxfordjournals.jbchem.a021519

[3] Liu CG, Xue C, Lin YH, Bai FW. Redox potential control and applications in microaerobic and anaerobic fermentations. Biotechnology Advances. Elsevier Inc.; 2013 p. 257–65. 10.1016/j.biotechadv.2012.11.005 23178703

[4] Sydow A, Krieg T, Mayer F, Schrader J, Holtmann D. Electroactive bacteria-molecular mechanisms and genetic tools. Appl Microbiol Biotechnol. 2014;98: 8481–8495. 10.1007/s00253-014-6005-z 25139447

[5] Watanabe K. Recent developments in microbial fuel cell technologies for sustainable bioenergy. J Biosci Bioeng. 2008;106: 528–536. 10.1263/jbb.106.528 19134546

[6] Rabaey K, Rozendal RA. Microbial electrosynthesis—revisiting the electrical route for microbial production. Nat Rev Microbiol. 2010;8: 706–716. 10.1038/nrmicro2422 20844557

[7] Flynn JM, Ross DE, Hunt KA, Bond DR, Gralnick JA. Enabling unbalanced fermentations by using engineered electrode-interfaced bacteria. mBio. 2010;1: 1–8. 10.1128/mBio.00190-10 PMC2975363 21060736

[8] Matsuda S, Liu H, Kato S, Hashimoto K, Nakanishi S (2011) Negative faradaic resistance in extracellular electron transfer by anode-respiring *Geobacter sulfurreducens* cells. Environ Sci Technol 45: 10163–10169. 10.1021/es200834b 22047596

[9] Ishii S, Suzuki S, Norden-Krichmar TM, Tenney A, Chain PSG, Scholz MB, et al A novel metatranscriptomic approach to identify gene expression dynamics during extracellular electron transfer. Nat Commun. 2013;4: 1601 10.1038/ncomms2615 23511466

[10] Matsuda S, Liu H, Kouzuma A, Watanabe K, Hashimoto K, Nakanishi S. Electrochemical gating of tricarboxylic acid cycle in electricity-producing bacterial cells of *Shewanella* . PLoS One. 2013;8: e72901 10.1371/journal.pone.0072901 23977370 PMC3748093

[11] Heidelberg JF, Paulsen IT, Nelson KE, Gaidos EJ, Nelson WC, Read TD, et al Genome sequence of the dissimilatory metal ion-reducing bacterium *Shewanella oneidensis* . Nat Biotechnol. 2002;20: 1118–1123. 12368813 10.1038/nbt749

[12] Kim BH, Kim HJ, Hyun MS, Park DH. Direct electrode reaction of Fe(III)-reducing bacterium, *Shewanella putrefaciens* . J Microbiol Biotechnol. 1999;9: 127–131.

[13] Ross DE, Flynn JM, Baron DB, Gralnick JA, Bond DR. Towards electrosynthesis in *Shewanella*: energetics of reversing the Mtr pathway for reductive metabolism. PLoS One. 2011;6: e16649 10.1371/journal.pone.0016649 21311751 PMC3032769

[14] Yang C, Rodionov DA, Li X, Laikova ON, Gelfand MS, Zagnitko OP, et al Comparative genomics and experimental characterization of N-acetylglucosamine utilization pathway of *Shewanella oneidensis* . J Biol Chem. 2006;281: 29872–29885. 16857666 10.1074/jbc.M605052200

[15] Pinchuk GE, Rodionov DA, Yang C, Li X, Osterman AL, Dervyn E, et al Genomic reconstruction of *Shewanella oneidensis* MR-1 metabolism reveals a previously uncharacterized machinery for lactate utilization. Proc Natl Acad Sci U S A. 2009;106: 2874–2879. 10.1073/pnas.0806798106 19196979 PMC2636740

[16] Rodionov DA, Yang C, Li X, Rodionova IA, Wang Y, Obraztsova AY, et al Genomic encyclopedia of sugar utilization pathways in the *Shewanella* genus. BMC Genomics. 2010;11: 494 10.1186/1471-2164-11-494 20836887 PMC2996990

[17] Scott JH, Nealson KH A biochemical study of the intermediary carbon metabolism of *Shewanella putrefaciens* . J Bacteriol 1994;176: 3408–3411. 8195102 10.1128/jb.176.11.3408-3411.1994 PMC205518

[18] Tang YJ, Meadows AL, Kirby J, Keasling JD Anaerobic central metabolic pathways in *Shewanella oneidensis* MR-1 reinterpreted in the light of isotopic metabolite labeling. J Bacteriol 2007;189: 894–901. 17114268 10.1128/JB.00926-06 PMC1797319

[19] Tang YJ, Hwang JS, Wemmer DE, Keasling JD *Shewanella oneidensis* MR-1 fluxome under various oxygen conditions. 2007;Appl Environ Microbiol 73: 718–729. 17098921 10.1128/AEM.01532-06 PMC1800752

[20] Howard EC, Hamdan LJ, Lizewski SE, Ringeisen BR. High frequency of glucose utilizing mutants in *Shewanella oneidensis* MR-1. FEMS Microbiol Lett. 2011;327: 9–14. 10.1111/j.1574-6968.2011.02450.x 22092702

[21] Choi D, Lee SB, Kim S, Min B, Choi I-G, Chang IS. Metabolically engineered glucose-utilizing *Shewanella* strains under anaerobic conditions. Bioresour Technol. 2014;154: 59–66. 10.1016/j.biortech.2013.12.025 24384311

[22] Newton GJ, Mori S, Nakamura R, Hashimoto K, Watanabe K. Analyses of current-generating mechanisms of *Shewanella loihica* PV-4 and *Shewanella oneidensis* MR-1 in microbial fuel cells. Appl Environ Microbiol. 2009;75: 7674–7681. 10.1128/AEM.01142-09 19837834 PMC2794086

[23] Kouzuma A, Meng X-Y, Kimura N, Hashimoto K, Watanabe K. Disruption of the putative cell surface polysaccharide biosynthesis gene SO3177 in *Shewanella oneidensis* MR-1 enhances adhesion to electrodes and current generation in microbial fuel cells. Appl Environ Microbiol. 2010;76: 4151–4157. 10.1128/AEM.00117-10 20453127 PMC2897461

[24] Kovach ME, Elzer PH, Hill DS, Robertson GT, Farris MA, Roop RM, et al Four new derivatives of the broad-host-range cloning vector pBBR1MCS, carrying different antibiotic-resistance cassettes. Gene. 1995;166: 175–176. 8529885 10.1016/0378-1119(95)00584-1

[25] Saltikov CW, Newman DK. Genetic identification of a respiratory arsenate reductase. Proc Natl Acad Sci U S A 2003;100: 10983–10988. 12939408 10.1073/pnas.1834303100 PMC196913

[26] Kouzuma A, Hashimoto K, Watanabe K. Influences of aerobic respiration on current generation by *Shewanella oneidensis* MR-1 in single-chamber microbial fuel cells. Biosci Biotechnol Biochem. 2012;76: 270–275. 22313754 10.1271/bbb.110633

[27] Kouzuma A, Hashimoto K, Watanabe K. Roles of siderophore in manganese-oxide reduction by *Shewanella oneidensis* MR-1. FEMS Microbiol Lett. 2012;326: 91–98. 10.1111/j.1574-6968.2011.02444.x 22092340

[28] Kouzuma A, Oba H, Tajima N, Hashimoto K, Watanabe K. Electrochemical selection and characterization of a high current-generating *Shewanella oneidensis* mutant with altered cell-surface morphology and biofilm-related gene expression. BMC Microbiol. 2014;14: 190 10.1186/1471-2180-14-190 25028134 PMC4112983

[29] Romine MF, Carlson TS, Norbeck AD, McCue LA, Lipton MS. Identification of mobile elements and pseudogenes in the *Shewanella oneidensis* MR-1 genome. Appl Environ Microbiol. 2008;74: 3257–3265. 10.1128/AEM.02720-07 18378659 PMC2394961

[30] Flores N, Xiao J, Berry A, Bolivar F, Valle F. Pathway engineering for the production of aromatic compounds in *Escherichia coli* . Nat Biotechnol. 1996;14: 620–623. 9630954 10.1038/nbt0596-620

[31] Flores S, Gosset G, Flores N, de Graaf AA, Bolívar F. Analysis of carbon metabolism in *Escherichia coli* strains with an inactive phosphotransferase system by ^13^C labeling and NMR spectroscopy. Metab Eng. 2002;4: 124–137. 12009792 10.1006/mben.2001.0209

[32] Hernández-Montalvo V, Martínez A, Hernández-Chavez G, Bolivar F, Valle F, Gosset G. Expression of *galP* and *glk* in a *Escherichia coli* PTS mutant restores glucose transport and increases glycolytic flux to fermentation products. Biotechnol Bioeng. 2003;83: 687–694. 12889033 10.1002/bit.10702

[33] Conway T. The Entner-Doudoroff pathway: history, physiology and molecular biology. FEMS Microbiol Rev. 1992;9: 1–27. 1389313 10.1111/j.1574-6968.1992.tb05822.x

[34] Lanthier M, Gregory KB, Lovley DR. Growth with high planktonic biomass in *Shewanella oneidensis* fuel cells. FEMS Microbiol Lett. 2008;278: 29–35. 17995953 10.1111/j.1574-6968.2007.00964.x PMC2228398

[35] Pinchuk GE, Geydebrekht OV, Hill EA, Reed JL, Konopka AE, Beliaev AS, et al Pyruvate and lactate metabolism by *Shewanella oneidensis* MR-1 under fermentation, oxygen limitation, and fumarate respiration conditions. Appl Environ Microbiol 2011;77: 8234–8240. 10.1128/AEM.05382-11 21965410 PMC3233039

[36] Bunch PK, Mat-Jan F, Lee N, Clark DP. The *ldhA* gene encoding the fermentative lactate dehydrogenase of *Escherichia coli* . Microbiology. 1997;143: 187–195. 9025293 10.1099/00221287-143-1-187

[37] Taylor BL, Zhulin IB. PAS domains: internal sensors of oxygen, redox potential, and light. Microbiol Mol Biol Rev. 1999;63: 479–506. 10357859 10.1128/mmbr.63.2.479-506.1999 PMC98974

[38] Brutinel ED, Gralnick JA. Preferential utilization of D-lactate by *Shewanella oneidensis* . Appl Environ Microbiol. 2012;78:8474–8476. 10.1128/AEM.02183-12 23001660 PMC3497373

[39] Aguilera L, Campos E, Giménez R, Badía J, Aguilar J, Baldoma L. Dual role of LldR in regulation of the *lldPRD* operon, involved in L-lactate metabolism in *Escherichia coli* . J Bacteriol. 2008;190: 2997–3005. 10.1128/JB.02013-07 18263722 PMC2293229

