# Supplementary material for: Metabolic Characteristics of a Glucose-Utilizing Shewanella oneidensis Strain Grown under Electrode-Respiring Conditions
Source: PLoS One. 2015 Sep 22;10(9):e0138813. doi: 10.1371/journal.pone.0138813 (PMC4579138; doi:10.1371/journal.pone.0138813)
Supplement: S1 Fig — MR-1 cells were cultivated in LMM supplemented with 15 mM D-lactate as the carbon and energy source or in a pyruvate minimal medium containing 15 mM pyruvate (in substitution for lactate in LMM) up to the early stationary growth phase. Results are expressed as relative values to mRNA levels in cells grown on pyruvate. Error bars represent standard deviation calculated from at least three measurements. (PDF) [file pone.0138813.s001.pdf]

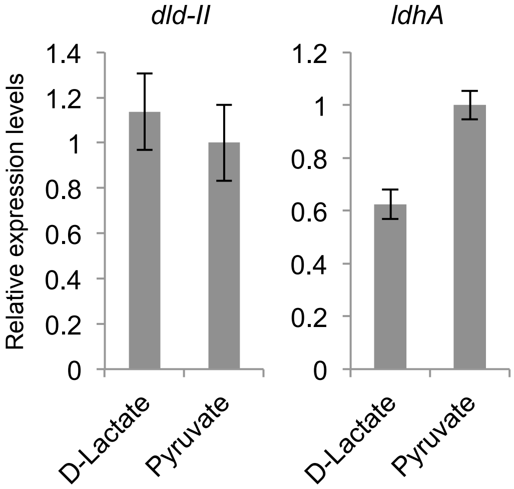

S1 Fig. qRT-PCR analyses of *dld-II* and *ldhA* in MR-1 cells grown with D-lactate and pyruvate
MR-1 cells were aerobically cultivated in LMM supplemented 15 mM D-lactate as the carbon and energy source or pyruvate minimal medium, which contained 15 mM pyruvate instead of the lactate in LMM, until the early stationary growth phase. Results are expressed as relative values to mRNA levels in the cells grown with pyruvate. The error bars represent the standard deviation calculated from at least three measurements.
