# Supplementary material for: Metabolic Characteristics of a Glucose-Utilizing Shewanella oneidensis Strain Grown under Electrode-Respiring Conditions
Source: PLoS One. 2015 Sep 22;10(9):e0138813. doi: 10.1371/journal.pone.0138813 (PMC4579138; doi:10.1371/journal.pone.0138813)
Supplement: S1 Table — (PDF) [file pone.0138813.s002.pdf]

S1 Table. Primers used in this study

Primer	Sequence (5' to 3')	Modification, for use
glk-F-KpnI	CGCGGTACCGACTTTAGCGGAGCAGTTGA	KpnI, pBBR-*glk-gal*P construction
glk-R-XhoI	CGGCTCGAGGATTTACAGAATGTGACCTAAG	XhoI, pBBR-*glk-gal*P construction
galP-F-XhoI	CCGCTCGAGCCATATTGGAGGGCATCATG	XhoI, pBBR-*glk-gal*P construction
galP-R-PstI	GACCTGCAGCTTGGGGAGATTAATCGTG	PstI, pBBR-*glk-gal*P construction
qRT-lldF-F	CGACCTACAGCGCCTTCTAC	qRT-PCR for *lldF*
qRT-lldF-R	AGTGTTAAGGCAGCCACCAC	qRT-PCR for *lldF*
qRT-dld-F	CATCGGCACTCAACTTCTCA	qRT-PCR for *dld-II*
qRT-dld-R	CGCAGGTATCAATCACATCG	qRT-PCR for *dld-II*
qRT-ldhA-F	GACCCCTACCCTAATCCAGC	qRT-PCR for *ldhA*
qRT-ldhA-R	GTTATCGGGTGTTAACGGGC	qRT-PCR for *ldhA*
qRT-pykA-F	CAAAATCGTCACCACACTGG	qRT-PCR for *pykA*
qRT-pykA-R	TGAGTAGCGCGTTTGAGATG	qRT-PCR for *pykA*
qRT-eda-F	TGCGCTTAAGGCTTTCTCTG	qRT-PCR for *eda*
qRT-eda-R	AATCCAGCTGCCACCAATAC	qRT-PCR for *eda*
qRT-aceF-F	TCATGAAGGAAGACGTGCAG	qRT-PCR for *aceF*
qRT-aceF-R	ACTTTAGGTGCCGCAATCAC	qRT-PCR for *aceF*
qRT-pflB-F	CCCAATGGTTGTGGGTAAAC	qRT-PCR for *pflB*
qRT-pflB-R	AGTGATTGGGTCTGCTTTGG	qRT-PCR for *pflB*
qRT-pta-F	TAGGTGCCCTGCTGCTTACT	qRT-PCR for *pta*
qRT-pta-R	GAGGTTTGCCAAGTGTTGGT	qRT-PCR for *pta*
qRT-16S-F	AGCGCAACCCCTATCCTTAT	qRT-PCR for 16S rRNA gene
qRT-16S-R	CGTAAGGGCCATGATGACTT	qRT-PCR for 16S rRNA gene
dld_5-O-SpeI	CTACACTAGTTAGCAAGGCTGAGCATACGA	SpeI, *dld-II* disruption
dld_3-O-SpeI	TGCTACTAGTTACTGTTGCGCCACTCAAAG	SpeI, *dld-II* disruption
dld_5-I	AACTTTGGTACCGATCACAGTGCAGCCGCTAAAACACT	Linker, *dld-II* disruption
dld_3-I	GTGATCGGTACCAAAGTTCGTCACTGGACGATCACCTA	Linker, *dld-II* disruption
ldhA_5-O-SpeI	CTAGACTAGTATCACCACCCGTGATAAAGC	SpeI, *ldhA* disruption
ldhA_3-O-SpeI	GCATACTAGTGCTCTTTTAGACGAGCCATC	SpeI, *ldhA* disruption
ldhA_5-I	AGCGCCGTTGGTACCTGAGTCGTAGTGTTTTGCGCT	Linker, *ldhA* disruption
ldhA_3-I	TCAGGTACCAACGGCGCTATGTGCAAGCAGTACTCG	Linker, *ldhA* disruption
